# Coping with tuberculosis and directly observed treatment: a qualitative study among patients from South India

**DOI:** 10.1186/s12913-016-1545-9

**Published:** 2016-07-19

**Authors:** Vijayashree Yellappa, Pierre Lefèvre, Tullia Battaglioli, Devadasan Narayanan, Patrick Van der Stuyft

**Affiliations:** Institute of Public Health, #250, 2nd C Main, 2nd C Cross, Girinagar I Phase, Bangalore, 560 085 Karnataka India; Institute of Tropical Medicine, Nationalestraat, 155, 2000 Antwerp, Belgium; Public Health Department, Faculty of Medicine, Ghent University, Ghent, Belgium

**Keywords:** Tuberculosis, Public-private mix, Coping, Finance, DOTS, Patient-centered care, Nutrition, Private practitioners, India, RNTCP

## Abstract

**Background:**

In India, the Revised National TB control programme (RNTCP) offers free diagnosis and treatment for tuberculosis (TB), based on the Directly Observed Treatment Short course (DOTS) strategy. We conducted a qualitative study to explore the experience and consequences of having TB on patients enrolled in DOTS and their caretakers in Tumkur district, located in a southern state of India, Karnataka.

**Methods:**

We conducted 33 in-depth interviews on a purposive sample of TB patients from three groups: (1) patients who reached RNTCP directly on their own and took DOTS at RNTCP; (2) patients who were referred by private practitioners (PPs) to RNTCP and took DOTS at RNTCP; and (3) patients diagnosed by RNTCP and took DOTS from PPs. Data was analyzed using a thematic approach with the support of NVivo9.

**Results:**

The study revealed that TB and DOTS have a large impact on patient’s lives, which is often extended to the family and caretakers. The most vulnerable patients faced the most difficulty in accessing and completing DOTS. The family was the main source of support during patient’s recovery. Patients residing in rural areas and, taking DOTS from the government facilities had to overcome many barriers to adhere to the DOTS therapy, such as long travelling distance to DOTS centers, inconvenient timings and unfavorable attitude of the RNTCP staff, when compared to patients who took DOTS from PPs. Advantages of taking DOTS from PPs cited by the patients were privacy, flexibility in timings, proximity and more immediate access to care. Patients and their family had to cope with stigmatization and fear and financial hardships that surfaced from TB and DOTS. Young patients living in urban areas were more worried about stigmatisation, than elderly patients living in rural areas. Patients who were referred by PPs experienced more financial problems compared to those who reached RNTCP services directly.

**Conclusion:**

Our study provided useful information about patient’s needs and expectations while taking DOTS. The development of mechanisms within RNTCP towards patient centered care is needed to enable patients and caretakers cope with disease condition and adhere to DOTS.

**Electronic supplementary material:**

The online version of this article (doi:10.1186/s12913-016-1545-9) contains supplementary material, which is available to authorized users.

## Background

In India, Tuberculosis (TB) remains a major public health problem and accounted for 24 % of the total 9 million cases reported worldwide in 2013 [1]. Following the World Health Organization (WHO) declaring TB as a ‘global emergency’ in 1993, the Government of India (GoI) launched the Revised National TB Control Programme (RNTCP) in 1997 based on WHO endorsed Directly Observed Treatment Short course (DOTS) strategy. RNTCP was expanded in a phased manner to achieve complete population coverage by 2006, with an overarching objective to detect at least 70 % of sputum positive pulmonary TB cases and to achieve a cure rate of at least 85 % [2]. The programme follows a ‘passive case finding’ approach, which assumes that people are able to recognize their symptoms and seek health care. Patients can avail RNTCP services either by directly accessing public health facilities or by being referred by private practitioners (PPs).

RNTCP is implemented mainly through public sector health facilities, which provide free diagnostic and treatment services. However in India, for many TB patients, the first point of contact for health care are PPs [3–6]. Recognizing this, the Government involved PPs through WHO recommended PPM (Public Private Mix)-DOTS strategy [7, 8]. This partnership enables PPs to refer patients to the RNTCP for either free diagnosis or treatment. Thereafter, the patient can either continue treatment within RNTCP or alternatively PPs themselves can provide DOTS.

Studies from India [9, 10] and elsewhere [11–14] have shown that accessing care and adhering with the long course of TB treatment is a complex phenomenon. However, most of these studies used a quantitative approach without further in-depth exploration into the “whys” of the issues. Few studies have used a qualitative approach to explore patient’s experiences during the different phases of DOTS treatment [15–18] Moreover, in India there are very few published qualitative studies [19, 20] that have captured the experience of patients and their caretakers in obtaining and completing DOTS.

WHO’s post 2015 TB strategy states “Integrated, patient centred care”, as one of the main component of the strategy [21]. Studies which analysed the reasons for non-adherence to TB treatment in RNTCP have concluded that it stems in a poor match between patients’ and programme’s needs and priorities [22]. To fulfil the aspiration of ‘patient centred care’, the programme managers and policy makers will have to give due attention to patient’s needs and expectations from the programme. With this background, we conducted this study to explore the experience and consequences of having TB on patients enrolled in DOTS and their caretakers in Tumkur district, located in a southern state of India, Karnataka. The study findings described in this paper are part of a larger study, which investigated how patients navigate through the health system and gain access to RNTCP and the organisational and operational challenges encountered in establishing collaboration between PPs and the RNTCP in Karnataka state.

## Methods

### Study design

Qualitative research using in-depth interviews as data collection tool.

### Setting

The study was conducted in Tumkur district (total population 2.716.997), Karnataka state, South India. The district is spread over 10,597 square kilometers, with a population density of about 253 persons per square kilometer. Like elsewhere in India, the district has a pluralistic health system composed of private and public health facilities. As per the RNTCP guidelines, Tumkur district is divided into six TB units, each catering to a population of 500,000 and responsible for program implementation. Under each TB unit, Designated Microscopy Centers (DMCs) provide free sputum microscopy services, each catering to a population of 100,000. Laboratory registers maintained at DMCs capture patient’s information such as name, age, sex, address, source of referral, date of sputum examination, and date of initiation of the TB treatment under the RNTCP. The district has 2,555 DOTS centers that provide free TB drugs and try to ensure treatment completion. The DOTS provider is an observer other than a family member, who supervises and supports the patients in taking their drugs at right doses and intervals. The DOTS provider can be either a public health facility (all of them provide DOTS), a PP, a health worker or any trained community volunteer who is acceptable and accessible to patients and accountable to the health system. If the patient resides far from the health facility, DOTS provision is made closer to the patient’s residence through a community health volunteer or a health worker.

### Recruitment of study participants

We conducted a total of 33 in-depth interviews on a purposive sample of new TB patients. Patients were recruited from three different groups that were defined for the purposes of the larger study mentioned above: (1) patients who reached RNTCP directly on their own and took DOTS at RNTCP; (2) patients who were referred by PPs to RNTCP and took DOTS at RNTCP; and (3) patients who were diagnosed by RNTCP and took DOTS from PPs, irrespective of how they initially reached RNTCP. Patients were further stratified for rural (*n* = 18) and urban (15) settings.

We aimed to interview eight respondents from each category. Patients were shortlisted from RNTCP laboratory registers. Patient characteristics such as, whether they reached RNTCP directly or were referred by a PP or taking DOTS from a PP, duration of the treatment, etc., were cross-checked with RNTCP TB registers and treatment cards. To reduce the recall bias, we selected patients who had completed their treatment in the last three months or who were about to complete the treatment in the next one month at the time of the interview. Although we targeted eight respondents in each category, we interviewed more respondents in the category of ‘referred by PPs’ (*n* = 17). This is because, in the course of data collection we discovered that some patients who were categorized as ‘having reached RNTCP directly’ had initially consulted PPs for their illness and were referred by them to the RNTCP.

Of the 33 interviews, 23 participants chose to be interviewed at their residence. Caretakers were present in 21 of these interviews, and helped patients reconstruct their experiences of care seeking. Of the 47 shortlisted patients, 14 patients could not be interviewed, 10 refused to be interviewed, and four had shifted their place.

### Data collection

Data was collected from May 2013 to August 2013. Three interviews guides, one for each category of patients (see Additional files 1,2, and 3), were pilot-tested in February 2013 and fine-tuned accordingly. In order to elicit quality data, interviews were preceded by warm up visits to interviewees by a field coordinator, who shared the information brochure and explained about the research project in the local language, Kannada. Efforts were made to build rapport with patients and an appointment was sought for interviews. Participants were given the choice to choose a date, time and place which was convenient for them. The Principal Investigator (PI) conducted the in-depth interviews in local language Kannada, which lasted between 45 min to one and half an hour. The main themes that were explored in the interviews were effects and consequences of having TB on individual and caregivers, and their experiences of completing DOTS therapy in RNTCP.

### Data analysis

A preliminary data analysis was conducted simultaneously with data collection. All interviews were audio recorded and transcribed verbatim by professional transcribers. Each transcription was then crosschecked by the principal investigator for accuracy and to include non-verbal communication. We combined deductive and inductive approaches to analyse the data. The deductive approach was based on the research questions and themes related to the experience of TB patients and their caretakers with the organizational and technical aspects of the delivery of DOTS, that were contained in the interview guide (e.g. previous and present experience with TB and DOTS, cost of the treatment, provider related issues, side effects). However, we kept the flexibility of including new themes emerging from the data (inductive approach).

**Fig. 1:**
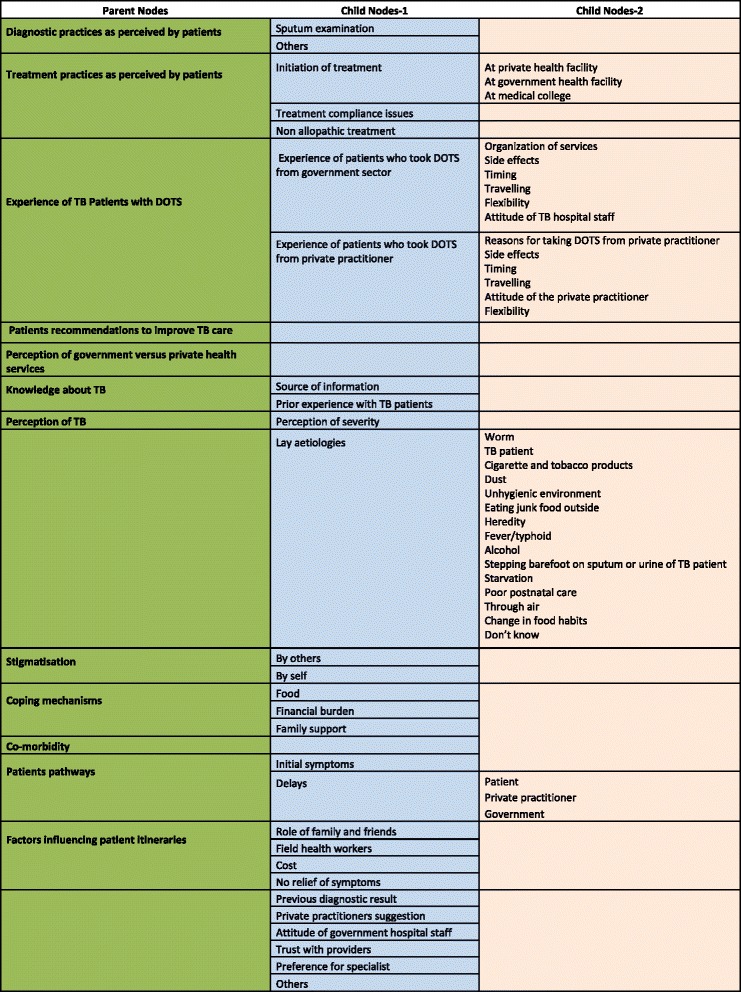
Coding Tree

We conducted a thematic analysis [23, 24]. A coding scheme was developed and refined progressively over time (Fig. 1). Segments of the transcripts were classified in the deductive themes. Simultaneously, themes that emerged through scrutinizing the data (inductive) were added. We systematically checked across the transcripts for the consistency of these emerging categories. In later stages of the analysis we explored the relationships between the themes and across different categories of participants to identify patterns in the data. The analysis was conducted with the support of QSR NVivo 9 (QSR International Pvt LTD, Melbourne, Australia).

To increase the internal validity of the analysis, the coding scheme (Fig. 1) was regularly discussed with a sociologist (PL) not involved in data collection and other members of the research team composed of researchers from different backgrounds (e.g. epidemiology, public health).

### Ethics

The study proposal received approval from the Institutional Ethics Committee of the Institute of Public Health, Bangalore and the Institutional Review Board of the Institute of Tropical Medicine, Antwerp and Institutional Ethics Committee of Antwerp University. To avoid any breach in medical confidentiality, the PI after line listing the potential TB patients, shared the list with the district TB officer, who in turn contacted field RNTCP staff to check with patients if they agreed to be interviewed. Following which, RNTCP staff contacted patients over phone and informed them about the interviews. RNTCP staff provided contact details of patients who agreed to participate and helped the PI locate patients using local maps. After explaining the confidentiality and nature of the research project in local language, written informed consent was obtained from all the participants by the PI for their participation in the research. Authorization for audio recording the interviews was also sought. Personal details of the participants were removed from the transcripts to ensure confidentiality and the audio files were anonymised. The NVivo database was password protected and was only accessible to the research team.

## Results

Our results are presented as follows: after summarising the characteristics of the patients, we describe the experience and consequences of having TB on patients enrolled in DOTS and their caretakers.

### Participant characteristics

The average age of participants was 40 with the youngest being 16 and the eldest 70. They were balanced for gender. Income per month ranged from USD 62 to USD 314. Of the 33 patients, three patients had extra pulmonary TB. All patients were new TB cases, except one and none of them took treatment for multidrug resistant TB. We are unable to provide a table of patient characteristics as the table could lead to conflict in protecting patients’ anonymity and confidentiality.

### Experience and consequences of having TB on patients enrolled in DOTS and their caretakers

As explained above, the recruited participants had recently completed their treatment or were by the end of it. Therefore, the experience of the patients with TB and their caretakers is indissolubly linked to their experience with DOTS.

We observed that having TB was not solely an individual patient problem, but in most cases a family issue. Some patients were found more vulnerable than others, because of the relative level of poverty and social support they possessed. Patients who were referred by PPs, patients belonging to lower Hindu caste, and patients with low and middle-income status, tend to speak more about their experiences. This is probably because they had to overcome more barriers to access and complete the DOTS treatment compared to the patients from higher Hindu caste and residing in urban areas.

Our data revealed that the most challenging issues for patients and their caretakers were related to coping with DOTS strategy itself. These challenges can be grouped in two broad categories: 1) organizational and technical aspects of the delivery of the service; and 2) socioeconomic and cultural implications of having a TB patient in the family. The latest encompasses issues that emerged through inductively analysing the data.

#### Organisational and technical aspects of the delivery of DOTS

Most of the respondents knew about the importance of completing the treatment and the need for regular follow-ups. They mentioned that the treatment was free under RNTCP and that they had to reach DOTS centres to swallow tablets on alternate days (i.e. Monday, Wednesday and Friday).

DOTS centres open only between 9 am to 1 pm, and patients and their caretakers had to make adjustments in their daily routines to reach the health facility. For instance, a family that lived on selling vegetables in the market found it difficult to continue their business, as they had to accompany their mother to a DOTS centre. Hence, they sacrificed certain profit by giving away the vegetables to middlemen in the market. A patient’s wife who was working as a sweeper in a theatre was the sole bread earner of the family and she struggled to take care of her husband and simultaneously attend her work. She explains: “*In the theatre I worked from morning 11.30 to 12.30 and then took my husband to the hospital by afternoon at 1’o clock to swallow tablets. Again from there we would start and reach home by 4.30. And again by 5’o clock, I used to rush to the theatre to work. Like this I managed*” [wife of patient who directly reached, M, R, 48 years].

Another concern highlighted by some patients was the need to arrange DOTS closer to the patient’s residence. Some patients, particularly the daily wage workers, had to forfeit their wages to reach the DOTS centre. Thus, they were left with a choice either to earn their livelihood or to take DOTS therapy. Patients, who were severely weak, who did not have stamina to walk to the DOTS centres and could not afford auto fare to commute to DOTS centre, were adversely affected.

Patients repeatedly expressed that DOTS should be made flexible. In one case a mother, who herself was a DOTS provider was not allowed to give DOTS to her own daughter, who suffered from TB. That particular patient was told by RNTCP staff to visit the hospital, which was 1.5 km away to take the DOTS. The family wanted to hide the disease status from neighbours, as the patient was not married and as they were anxious that suffering from TB could become a threat to a future marriage. The mother expressed her disappointment and was hurt by RNTCP staff’s apathy: “*I pleaded them saying “please give it* [TB drugs] *to me. But they did not give. I felt very bad madam. I have given DOTS to so many patients*” [Mother of directly reached patient, F, R, 19 years].

Another patient cautioned, if the programme fails to provide the flexibility in DOTS, patients might quit treatment in the middle. An excerpt: “*for people who do daily wage work or go to garment industry, if they reach the workplace late by even 15 min, they will be asked many questions. Will they* [owner] *consider that they [patients] had been to take the medicines? No… they will be straightaway sent back home. For a patient, his work is more important than the tablet and if the authorities refuse to oblige, he will quit the tablets and proceed for his work*” [PP referred, M, U, 32 years].

Patients highlighted the need for improving the attitude of RNTCP staff. They suggested that the program staff should proactively know patient’s job profile and prioritise those patients who go for work while giving DOTS. This would enable patients to go back to their work after taking tablets. One patient narrated: “*He* [another patient who came to take DOTS] *is only 35 years old and has two small children. He comes to TB hospital by morning 9.30 and by the time he reaches his house it will be afternoon 2’o clock. He will not have any time to go to work. He is in such a situation that if he does not go for his treatment, he will die and if he does not go for work, his children will die*” [PP referred, M, U, 65 years].

Almost all patients experienced severe vomiting and giddiness in the initial stages of the treatment and also expressed the need for patient counselling especially in the initial stages of DOTS. Some patients were unhappy that, RNTCP staff attitude was not good in such situations, when patients required the maximum support from them. An excerpt: “*If a patient gets vomiting sensation after taking tablets, TB centre staff should take care of the patient. But these people just ask the patient to go away and vomit. So, some patients because of vomiting sensation associated with TB tablets don’t take the tablets*” [PP referred, M, U, 65 years].

Contrastingly, patients who took DOTS from PPs did not face any such difficulties and all of them were satisfied with DOTS provided by PPs. All the eight patients who received DOTS from PPs resided in urban areas. The common reasons cited for taking DOTS from PPs were the geographical proximity, and flexibility provided by PPs in timings to take DOTS as per the patient’s needs. Patients could consult PPs immediately when they experienced some side effects of tablets. One patient who was a sales representative narrated how convenient it was to take DOTS from a PP: *“Since I had to leave to my job by 9.00 am, the doctor made arrangements at my convenience. The nurse used to give me tablets early in the morning and I used to take tablets and leave for my job”* [Taken DOT from PP, M, U, 50 years].

Female patients were particularly happy about the privacy, which was provided at PP’s clinics while taking DOTS. An excerpt; “*I would have missed my college, if I had to go in the morning to take tablets from TB hospital. Moreover, there used to be so many people, there* [TB hospital]. *I used to feel shy. Here* [PP’s clinic] *no one will be there [she was shy while narrating this]. I used to feel comfortable here*” [Taken DOT from PP, F, U, 18 years].

Only in two cases, the RNTCP staff took the initiative to link them to the nearest PPs who provided DOTS. In the remaining cases, patients had to negotiate with the staff to look for a DOTS provider closer to their residence “*I kept on telling them that it is difficult for me to come here* [TB hospital] every day. *Then they said, we cannot give drugs to home, but we can give it to a doctor nearby my house. I told them to give tablets there* [PP clinic] *and I took there for six months continuously*” [Taken DOTS from PP, M, U, 43 years].

#### Socioeconomic and cultural implications of having a TB patient in the family

The main implications of having TB are fear and stigmatization and an economical burden. Like any disease TB has a cost for the patient. However, inductively analysing our data we found that DOTS as organized in India adds to these problems: the patient becomes visible to others and due to the long duration of the treatment, the families are economically affected.

Patients had mixed feelings when they were diagnosed with TB. They felt sad, shocked and afraid. Patients, especially those whose relatives had died of TB in the past perceived TB as a big problem. About one third of patients felt that TB was not a dangerous disease and that, if the treatment is taken regularly, it could be cured like any other disease. Five of these patients had prior exposure to TB patients who were cured.

Few patients were relieved when they were diagnosed with TB, because they perceived initial symptoms such as coughing blood as more serious or they were afraid to be diagnosed with HIV or cancer. However, these results should be interpreted with caution, because the perception of severity changed with time in some patients due to (a) the counselling from doctors and paramedical staff during the course of treatment (b) moral support provided by friends and relatives and (c) relief of symptoms after starting the treatment.

We observed that almost all the respondents were uncomfortable to use the word “TB”. Instead, they referred to it as ‘illness’. Most patients did not share their disease status with their relatives or neighbours for the fear of getting excluded or getting bad comments. An excerpt: “*I did not want our neighbors to know about this. They* [TB staff] *said, “we can give the tablets to Anganawadi Centre* [DOTS center] *or somebody’s house near my residence”. We refused and said, we will not go anywhere and we will come swallow tablets in the hospital itself*” [Directly reached, F, R, 19 years].

The tendency of hiding the disease was more among younger patients. Contrastingly, elderly patients in the rural areas tend to share their difficulties with neighbours and there was a certain degree of social acceptance for these patients. We also observed that patients who had social support in the rural areas were less worried about stigmatisation compared to those who did not have. However, patients residing in urban areas took utmost care not to reveal their disease status to anybody, irrespective of the age group. In rural areas, neighbours usually knew the disease status, as it was difficult to hide from the neighbours as expressed by one patient: *“Normally village people will ask lot of questions, whereas in cities no one will ask. When people ask, we have to give reasons as to why we are visiting the hospital daily. Otherwise they will keep asking the same question another 20 times. A situation will come, when we will have to tell everyone whoever asks”* [PP referred, F, R, 30 years].

Many patients experienced discrimination in one form or the other. In many instances, patient’s relatives stood far from them or did not allow children near them for the fear of contracting the disease. Patients were hurt by this discrimination. One patient explains, “*when my relatives used to come home to see me, they used to hesitate to even drink water in my home*” [PP referred, M, U, 41 years]. However, the same patient defends their behaviour, explaining that TB is a contagious disease and it is quite normal for relatives to fear about it and behave in such a manner. “*It is just similar to situations like, if we have dengue in our surroundings, we tend to shut the doors to get rid of the mosquitoes*”.

Immediate family members were always informed about the disease. In almost all cases, the family was the main source of support during patient’s recovery. However, patients imposed certain behaviour on themselves such as self-isolation, keeping their plates and cups separately, not allowing children to come close to them, etc. This behaviour was mostly ‘*self-induced*’ because of the fear of spreading the disease to family members and they were extremely cautious about it. Most patients knew that TB is contagious, and hence closed their mouth while coughing to prevent the spread of the disease to other family members. However, this fear came down eventually when they saw many patients taking DOTS in hospitals. These ‘self-induced’ behaviours were influenced by previous exposure of patient’s parents and their relatives to TB and also induced by health staff suggestions. An excerpt:

“*The doctor had informed that the disease would not spread after two months. It will not have the strength. Hence after two moths I went out of house to meet friends”* [PP Referred, Male (M), Urban (U), 32 years].

We observed that there was a strong belief among the caretakers that TB patients should be isolated and be provided with separate plates and bed covers. In two cases, it was apparently advised by health staff. Many caretakers believed washing patients clothes in hot water will help in speedy recovery: “*clothes should be washed in hot water everyday. Then it will be cured fast”* [Directly reached, F, R, 41 years]. One patient was made to stay in a separate house where cattle were kept and food was served there till 4–5 months after the initiation of treatment. That particular patient narrates how painful this isolation was: “*I was feeling very bad that I had to stay away like this. I was thinking why I should live like this, when nobody wants me. But day-by-day I was improving and I was cured. I forgot about all those things*” [PP referred, M, R, 50 years].

Patients and their family struggled to meet the financial burden that surfaced due to TB. All patients suffered from severe symptoms, which incapacitated their ability to work, and found it difficult to earn their living. Major expenditure was towards arranging nutritious food and transportation costs to reach DOTS centre on alternate day. Patients who could afford were mostly residing in the urban areas. They travelled to the DOTS centre in a motorcycle or an auto, mostly accompanied by a caretaker. In India, each trip costs a minimum of Rs.60 [1USD], which is costly considering the fact that patients were unable to earn income during the period. Patients and caretakers from the rural areas took loans to meet the additional expenses from relatives, friends, banks, self-help groups, working place and pledge the jewellery and house. In some instances, parents tried to earn extra money working as maids to take care of their offspring who suffered from TB. Ten patients had taken loans and nine out of them were referred by PPs. They repented on their decision to seek care from PPs instead of directly reaching RNTCP services. Many were still repaying the loan at the time of the interview. An excerpt “*If I had gone to government hospital in the first instance itself, we would not have taken this much loan. We would not have been a debtor. God knows when I will be able to clear my loans*” [PP referred, M, R, 50 years].

Patients who had severe symptoms were admitted to the hospital and so found it hard to manage their business or secure their job during hospitalisation. One patient, who worked as an electric supervisor, could not stop working for the fear of losing his job. He left for his work from the hospital without informing anybody, with the intravenous cannula intact in his hand. He narrated:

“Even when I was admitted, I had removed the drips [intra venous fluids] and went to work without the doctor’s and family’s notice. If I did not go, then I would have lost the job and I would not have survived” [PP referred, M, U, 41 years]

Another issue that increased the economic burden of the families was the strong belief that existed among patients and caregivers that it was mandatory to eat non-vegetarian food for speedy and complete recovery from TB. Thus, patients spent substantial amounts of money towards arranging non-vegetarian food. Caretakers on the other hand, made efforts to cope up with extra financial burden to meet this requirement. Villagers went to town to buy fruits and some caregivers managed to cook meat once in three days with lots of financial difficulties and, in some instances neighbours shared mutton dishes when they prepared. Hence, some respondents suggested that the government should provide either banana or egg to the patients along with DOTS.

Two patients, who were Hindus, narrated the experience of eating beef in the anticipation of fast recovery from TB. Eating beef is not allowed in Hindu religion and these two respondents revealed this fact only towards the end of interview with lot of hesitation. Since beef could not be cooked at home, they had to go in search of hotels to eat beef. Every beef meal costed them USD 2–3. They firmly believed that eating beef helped them in fast recovery and rapid weight gain. They took utmost care not to reveal it to anybody, including the family members. “*If we eat fish and beef along with tablets properly, we will get cured fast. Only mere tablets and eating the usual food prepared at home will not cure TB*” [PP referred, M, U, 41 years].

None of the interviewed patients paid money to avail RNTCP services in the Government hospitals except few who had purchased streptomycin injection from private pharmacies, due to the stock out in government facilities. Almost all the participants expressed gratitude towards government for delivering them free medications, but also suggested that the Government should provide financial assistance to the poor patients who could not earn their livelihood while taking DOTS. Some suggested provision of free shelter and food in the initial two months of the treatment for severely ill, and poor patients, with no social support.

## Discussion

This qualitative study has revealed that TB and DOTS have a large impact on patient’s lives, which is often extended to the family and caretakers. The patients who faced the most difficulty in accessing and completing DOTS were the ones who were most vulnerable to the disease because of the relative level of poverty and social support they possessed. In all cases, the family was the main source of support during patient’s recovery. Patients residing in rural areas, taking DOTS from the government hospitals had to overcome many barriers to adhere to the DOTS therapy, such as long travelling distance to DOTS centers, inconvenient timings and unfavorable attitude of the RNTCP staff, when compared to patients who took DOTS from PPs in urban area. Patients identified advantages of taking DOTS from PPs such as privacy, flexibility in timings, proximity and more immediate access to care. Patients and their family had to cope with stigmatization and fear and financial hardships that surfaced from TB and DOTS. Patients who were referred by PPs experienced more financial struggle compared to those who reached RNTCP services directly.

Qualitative findings are not generalizable outside study settings. However, our findings are most probably transferable to similar settings in India. Though we took steps to reduce recall bias during participant’s recruitment, it could not be ruled out completely. The results described in the present study are solely based on patient’s and caregiver’s experience and reconstruction of the events that occurred during the treatment of the illness. It could have been useful to complement this information with the perspective of health care providers. Patients were identified from the RNTCP registers and were initially contacted by RNTCP staff over phone to participate in the study. Hence, some respondents could have not wanted to share information regarding program staff that could have jeopardized their relationship with them.

Lienhardt and Ogden have argued that strict and universal application of DOTS can decrease the effectiveness of TB control [25]. Our respondents also expressed concerns for introducing more flexibility in the DOT and suggested to improve the attitude of DOTS providers as reported in other studies [26]. In their study Pranaya Mishra et al. [27] concluded that poor communication between patients and DOTS providers were significantly associated with non-adherence. Our results and the above studies call for strengthening the competences of the health personnel involved in TB care and to improve communication through appropriate training.

In our study, we observed that, patients who took DOTS from PPs were satisfied with the flexibility of the service and the care provided by them. This supports a study finding that patients who took TB treatment outside RNTCP perceived DOTS, as applied in India, to be rigid and intrusive [28]. As mentioned in the background, the majority of TB patients in India seek care from PPs. Hence, every effort to control TB should prioritize the ways to engage with PPs optimally, including the provision of DOTS. In our study, it was found that the programme took the initiative to link only two patients with PPs for DOTS provision. This indicates the need to involve more PPs optimally in the delivery of DOTS, regardless of whether patients seek care in the public or the private sector.

There has been an extensive debate around DOTS versus Self-Administered Therapy (SAT) for TB patients. Results of two randomized control trials have shown that there was little to be gained by DOTS compared with SAT [29, 30]. A recent study from India also concluded that there was no significant difference between success rate in patients taking DOTS and SAT [31]. Similar findings were obtained in an integrated review of DOTS conducted in Latin-America [32]. Some alternatives for DOTS administration have been also studied and promoted such as community-based DOTS. A systematic review recently conducted by Zhang and colleagues [33] on the impact of community-based DOTS showed that this alternative has improved TB treatment outcomes. Volminck and colleagues, in their systematic review on DOTS for TB control concluded that health care delivery oriented towards the needs and preferences of patients seemed to lead to more satisfied patients [34]. Studies have shown that introducing flexibility in DOTS may indeed allow for more efficient [35, 36] and cost effective [37]. Recently, there have been moves in the international TB control landscape. WHO TB control, previously heavily based on DOTS [38] has shifted to the STOP TB Partnership, that focuses on a patient-centered approach to TB care taking into account the needs, perspectives, and individual experiences of people affected by TB [39].

It is essential that programme managers recognise that patients strive to adhere to their treatment, but structural barriers prevent them from doing so [40]. Our study resonates largely with the social determinants model of health which put emphasis on the social factors that could affect individuals’ ability to seek health care and adhere to a course of treatment [41, 42]. Currently RNTCP is vertically oriented, with more emphasis on programme targets than on the patients [43]. TB advocates have urged for a patient-centered solution to TB control, delivered with dignity and compassion [44]. RNTCP may have to consider this as a priority, if its ambitious goal envisaged in its national strategic plan of providing ‘universal access to quality assured free diagnosis and treatment to patients’, is to become a reality [2]. Being responsive to the local context, meeting the patient’s expectations and needs, and empower patients with the help of the family and caretakers to complete the treatment is a way forward [45, 46].

It was our consistent finding that patients always shared their disease condition with their close family members. We further observed that the adherence to treatment was heavily dependent on patient's and caretaker’s competing needs of everyday life. Similarly to other studies, social support from spouse and close family members was crucial in adhering to the DOTS therapy [47] and the more the social support, the better the coping with the disease condition [48]. Therefore, the TB programme needs to acknowledge the crucial role of the families and proactively involve them in patient counselling to increase their knowledge and awareness about the disease. A better understanding of TB as a disease condition within the families may improve patient care.

The majority of the respondents did not share their disease status with others, except the immediate family members. A study which assessed the overall TB stigma index in India, Bangladesh, Malawi and Colombia, reported that it was highest in India [49]. Studies from India and neighboring Pakistan reported that women were concerned about rejection by their husbands, harassment by in-laws, and the reduced chances of marriage because of TB [50–52] Similarly, we observed that women respondents in our study were satisfied receiving DOTS from PPs, as it provided them privacy. Patients were discriminated and many of them self-isolated with the fear that they will spread the disease to kith and kins, a finding similar to a qualitative study from Nepal [53]. In our study we found that some field health workers suggested self-isolation to some of the patients. This calls for training of this staff. Overall, all these findings point towards the stigma of TB in the Indian society and the urgent need for the programme to reduce stigma related problems in the TB care and control efforts, by conducting effective awareness programs. However, it is important to point out that the way DOTS is administered in India increases the stigmatization of the disease as shown by our findings.

Our study findings add to the evidence that TB patients in India incur large costs associated with TB illness [54–57]. TB had devastating socio-economic impact on our study participants and their families, especially from rural areas and further increased their poverty as reported in other studies [58, 59]. This phenomenon typically illustrates the ‘medical poverty trap’, a situation where expenditure increases and income decreases [60]. We found that arranging for ‘nutritious’ food during the course of the treatment created a significant financial burden, a finding similar to a study from Zambia which reported on substantive ‘special food’ expenditures [61].

Our study findings are also in line with the widely recognized phenomenon of ‘inverse care law’, which explains that those who are in most need of healthcare are least able to access services [62]. Because of long distance and inconvenient timings of DOTS centers, patients had to struggle to reach them on time. Studies have shown that long distance from DOTS centre, having an occupation or other socio-cultural obligations are some of the reasons for patients to discontinue TB treatment [47, 48]. Another study, which analyzed the factors affecting compliance of patients with DOTS, reported that it is affected by travel expenses and the amount of time that patients must spend travelling to treatment centres. Cost of transport was the reason most frequently given for non-compliance [63]. For improving TB control several authors have stressed that provision of DOTS is insufficient and called for introducing pro poor, equity enhancing measures to address poverty in TB control efforts [64–66]. Initiatives of systematically including TB patients under social protection schemes may prove beneficial in this regard [67].

## Conclusion

Our study provides useful information about patient’s needs and expectations regarding the delivery of DOTS in India. We also have flagged the need for developing mechanisms within RNTCP to help patients and their families to cope up with disease condition. The programme should make a paradigm shift, to move to a more patient centred approach.

## Abbreviations

DMCs, Designated Microscopy Centers; DOTS, Directly Observed Treatment Short course; GoI, Government of India; PI, Principal Investigator; PPM, Public Private Mix; PPs, Private Practitioners; RNTCP, Revised National TB control programme; SAT, Self-Administered Therapy; TB, Tuberculosis; WHO, World Health Organization

## Additional files

Additional file 1: Interview guide 1. Patients under PPM-DOTS. Questionnaire for in-depth interviews of TB patients under PPM-DOTS (DOCX 29 kb) Additional file 2: Interview guide 2. Patients referred by PP. Questionnaire for in-depth interviews of TB patients referred by Private Practitioners (PPs) (DOCX 29 kb) Additional file 3: Interview guide 3. Patients who reached RNTCP directly. Questionnaire for in-depth interviews of TB patients who have reached DMC directly (not referred by Private Practitioner) (DOCX 29 kb)

## References

[1] World Health organisation. Global TB Report. 2014. http://www.who.int/tb/publications/global_report/en/. Accessed 12 June 2015.

[2] Sachdeva KS, Kumar A, Dewan P, Kumar A, Satyanarayana S (2012). New Vision for Revised National Tuberculosis Control Programme (RNTCP): Universal access - “Reaching the un-reached.”. Indian J Med Res.

[3] Sachdeva KS, Satyanarayana S, Dewan PK, Nair SA. Source of Previous Treatment for Re-Treatment TB Cases Registered under the National TB Control Programme. PLoS Med. 2011;6(7). 10.1371/journal.pone.0022061 PMC3140992 21814566

[4] Kapoor SK, Raman AV, Sachdeva KS, Satyanarayana S (2012). How did the TB patients reach DOTS services in Delhi? A study of patient treatment seeking behavior. PLoS One.

[5] Hazarika I (2011). Role of private sector in providing tuberculosis care: evidence from a population-based survey in India. J Glob Infect Dis.

[6] Satyanarayana S, Nair SA, Chadha SS, Shivashankar R, Sharma G, Yadav S (2011). From where are tuberculosis patients accessing treatment in India? Results from a cross-sectional community based survey of 30 districts. PLoS One.

[7] Government of India. Revised Schemes for NGOs and Private Providers.2008. http://www.tbcindia.nic.in/showfile.php?lid=3143 Schemes NGO-PP.pdf. Accessed on 1 September 2014.

[8] WHO. Public private mix for TB care and control -A tool kit. WHO. 2010. http://www.who.int/tb/careproviders/ppm/PPMToolkit.pdf. Accessed on 10 Dec 2014.

[9] Charles N, Thomas B, Watson B, Raja Sakthivel M, Chandrasekeran V, Wares F (2010). Care seeking behavior of chest symptomatics: a community based study done in South India after the implementation of the RNTCP. PLoS One.

[10] Nimbarte SB, Wagh V, Selokar D (2011). Health seeking behaviour among pulmonary tuberculosis patients in rural part of central India. Int J Biol Med Res.

[11] Xu B, Diwan VK, Bogg L (2007). Access to tuberculosis care: what did chronic cough patients experience in the way of healthcare-seeking?. Scand J Public Health.

[12] Ukwaja KN, Alobu I, Nweke CO, Onyenwe EC (2013). Healthcare-seeking behavior, treatment delays and its determinants among pulmonary tuberculosis patients in rural Nigeria: a cross-sectional study. BMC Health Serv Res.

[13] Li Y, Ehiri J, Tang S, Li D, Bian Y, Lin H (2013). Factors associated with patient, and diagnostic delays in Chinese TB patients: a systematic review and meta-analysis. BMC Medicine.

[14] Makwakwa L, Sheu M, Chiang C, Lin S, Chang PW (2014). Patient and health system delays in the diagnosis and treatment of new and retreatment pulmonary tuberculosis cases in Malawi. BMC Infect Dis.

[15] Ten Asbroek AH, Bijlsma MW, Malla P, Shrestha B, Delnoij DMJ (2008). The road to tuberculosis treatment in rural Nepal: A qualitative assessment of 26 journeys. BMC Health Serv Res.

[16] Munro SA, Lewin SA, Smith HJ (2008). Patient adherence to tuberculosi treatment: a systematic review of qualitative research. Int J Tuberc Lung Dis.

[17] Hane F, Thiam S, Fall AS, Vidal L, Diop AH, Ndir M (2007). Identifying barriers to effective tuberculosis control in Senegal : an anthropological approach. Intern J Tuberc lung Dis.

[18] Hasker E, Khodjikhanov M, Sayfiddinova S, Rasulova G, Yuldashova U, Uzakova G (2010). Why do tuberculosis patients default in Tashkent City, Uzbekistan? A qualitative study. Int J Tuberc Lung Dis.

[19] Isaakidis P, Rangan S, Pradhan A, Ladomirska J, Reid T, Kielmann K (2013). I cry every day”: experiences of patients co-infected with HIV and multidrug-resistant tuberculosis. Trop Med Int Heal.

[20] Deshmukh RD, Dhande DJ, Sachdeva KS (2015). Patient and provider reported reasons for lost to follow Up in MDRTB treatment : a qualitative study from a drug resistant TB centre in India. PLoS One.

[21] World Health organisation. Global strategy and targets for tuberculosis prevention, care and control after 2015. http://www.who.int/tb/post2015_tbstrategy.pdf. Accessed on December 2015.

[22] Jaiswal A, Singh V, Ogden JA, Porter JDH, Sharma PP, Sarin R (2003). Adherence to tuberculosis treatment : lessons from the urban setting of Delhi. India Trop Med Int Heal.

[23] Boyatzis RE (1998). Transforming qualitative information: thematic analysis and code development.

[24] Mq P (2002). Qualitative Research and Evaluation Methods.

[25] Lienhardt C, Ogden JA (2004). Tuberculosis control in resource-poor countries: Have we reached the limits of the universal paradigm?. Trop Med Int Heal.

[26] Jain M, Chakole SV, Pawaiya AS, Mehta SC (2012). Knowledge, attitude and Practice of DOTS providers under RNTCP in Ujjain, Madhya Pradesh. Natl J community Med.

[27] Mishra P, Hansen HHP, Svend Sabore KKK (2006). Adherence to DOTS. Patient Educ Couns.

[28] Pinto LM, Udwadia ZF (2010). Private patient perceptions about a public programme; what do private Indian tuberculosis patients really feel about directly observed treatment ?. BMC Public Health.

[29] Walley JD, Khan MA, Newell JN, Khan MH (2001). Effectiveness of the direct observation component of DOTS for tuberculosis: A randomised controlled trial in Pakistan. Lancet.

[30] Newell JN, Baral SC, Pande SB, Bam DS, Malla P (2006). Family-member DOTS and community DOTS for tuberculosis control in Nepal: cluster-randomised controlled trial. Lancet.

[31] Parida A, Bairy KL, Chogtu B, Magazine R, Vidyasagar S (2014). Comparison of directly observed treatment short course with self-administered therapy in pulmonary tuberculosis in udupi district of southern India. J Clin Diagnostic Res.

[32] Zuniga JA (2015). An integrated review of directly observed therapy for tuberculosis in Latin America. Hisp Heal Care Int.

[33] Zhang H, Ehiri J, Yang H, Tang S, Li Y (2016). Impact of community-based DOT on tuberculosis treatment outcomes: a systematic review and meta-analysis. PLoS One.

[34] Volmink J, Garner P. Directly observed therapy for treating tuberculosis. Cochrane Data base Syst Rev. 2015;(5):DOI: 10.1002/14651858.CD003343. 10.1002/14651858.CD003343.pub4 PMC4460720 26022367

[35] Macq J, Solis A, Martinez G, Martiny P (2008). Tackling tuberculosis patients’ internalized social stigma through patient centred care: an intervention study in rural Nicaragua. BMC Public Health.

[36] Dermot Maher, Raj Gupta, Mukund Uplekar, Chris Dye MR. Directly Observed Therapry and Treatment adherence. Lancet. 2000;356(9234). 10.1016/S0140-6736(05)72652-0 11041426

[37] Prado TN, Wada N, Guidoni LM, Golub JE, Dietze R, Maciel EL (2011). Cost-effectiveness of community health worker versus home-based guardians for directly observed treatment of tuberculosis in Vitoria, Espirito Santo State Brazil. Cad Saude Publica.

[38] Uplekar M, Figueroa-Munoz J, Floyd K, M U, J F-M, K F, et al. The Stop TB Strategy: building on and enhancing DOTS to meet the TB-related Millennium Development Goals. WHO Rep. 2006;22.

[39] Stop TB Partnership. The Global Plan to End TB: The Paradigm Shift 2016–2020. 2015. Available from: http://www.stoptb.org/assets/documents/global/plan/GlobalPlanToEndTB_TheParadigmShift_2016-2020_StopTBPartnership.pdf

[40] Lambert ML, Van der Stuyft P (2005). Delays to tuberculosis treatment: shall we continue to blame the victim?. Trop Med Int Health.

[41] Hargreaves JR, Boccia D, Evans CA, Adato M, Petticrew M, Porter JDH (2011). The social determinants of tuberculosis: from evidence to action. Am J Public Health.

[42] Farmer P (1996). Social Inequalities and Emerging Infectious Diseases. Emerg Infect Dis.

[43] Singh V (1992). Significance of foreign funding in developing health programmes in India. The case study of RNTCP in the overall context of North–south co-operation. Heal Adm.

[44] Pai M, Yadav P, Anupindi R (2014). Tuberculosis control needs a complete and patient-centric solution. Lancet Glob Heal.

[45] Macq JCM, Theobald S, Dick J, Dembele M (2003). An exploration of the concept of directly observed treatment for tuberculosis patients : from a uniform to a customised approach. Int J Tuberc Lung Dis.

[46] Macq J, Torfoss T, Getahun H (2007). Patient empowerment in tuberculosis control: reflecting on past documented experiences. Trop Med Int Health.

[47] Lewis CP, Newell JN (2009). Improving tuberculosis care in low income countries - a qualitative study of patients’ understanding of “patient support” in Nepal. BMC Public Health.

[48] Marra CA, Marra F, Cox VC, Palepu A, Fitzgerald JM (2004). Factors influencing quality of life in patients with active tuberculosis. Health Qual Life Outcomes.

[49] Somma D, Thomas BE, Karim F (2008). Gender and socio-cultural determinants of TB-related stigma in Bangladesh, India, Malawi and Colombia. Int J Tuberc Lung Dis.

[50] Jaggarajamma, K,ramachandrn R,Nirups Charles,V Chandrasekaran, M Muniyandi S. Psycho-socialdysfunction: Perceived and enacted stigma among tuberculosis patients registered under Revised National Tuberculsois control programme. Indian J Tuberc. 2008;031(44). 19295104

[51] Nair DM, George A, Chack KT. Tuberculosis in Bombay : new insights from poor urban patients. 1997;12(1):77–85 10.1093/heapol/12.1.77 10166105

[52] Khan A, Walley J, Newell J, Imdad N (2000). Tuberculosis in Pakistan: Socio-cultural constraints and opportunities in treatment. Soc Sci Med.

[53] Baral SC, Karki DK, Newell JN (2007). Causes of stigma and discrimination associated with tuberculosis in Nepal: a qualitative study. BMC Public Health.

[54] Tanimura T, Jaramillo E, Weil D, Raviglione M, Lonnroth K (2014). Financial burden for tuberculosis patients in low- and middle-income countries: a systematic review. Eur Respir J.

[55] Pantoja A, Floyd K, Unnikrishnan KP, Jitendra R, Padma MR, Lal SS (2009). Economic evaluation of public-private mix for tuberculosis care and control, India. Part I. Socio-economic profile and costs among tuberculosis patients. Int J TB Lung.

[56] Floyd K, Arora VK, Murthy KJR, Lonnroth K, Singla N, Akbar Y (2006). Cost and cost-effectiveness of PPM-DOTS for tuberculosis control : evidence from India. Bull World Health Organ.

[57] John KR, Daley P, Kincler N, Oxlade O, Menzies D (2009). Costs incurred by patients with pulmonary tuberculosis in rural India. Intern J Tuberc lung Dis.

[58] Rajeswari R, Balasubramanian R, Muniyandi M, Geetharamani S, Thresa X, Venkatesan P (1999). Socio-economic impact of tuberculosis on patients and family in India. Intern J Tuberc lung Dis.

[59] Mauch V, Woods N, Kirubi B, Kipruto H, Sitienei J, Klinkenberg E (2011). Assessing access barriers to tuberculosis care with the tool to Estimate Patients’ Costs: pilot results from two districts in Kenya. BMC Public Health.

[60] Dahlgren G, Whitehead M (2006). European strategies for tackling social inequities in health: Levelling up Part 2. World Heal Organ.

[61] Needham DM, Bowman D, Foster SD, Godfrey-Faussett P (2004). Patient care seeking barriers and tuberculosis programme reform: a qualitative study. Health Policy.

[62] Hart JT (1971). The Inverse Care Law. Lancet.

[63] Boyle SJO, Power JJ, Ibrahim MY, Watson JP (2002). Factors affecting patient compliance with anti-tuberculosis chemotherapy using the directly observed treatment, short-course strategy. Int J Tuberc Lung Dis.

[64] Banerji D, Virchow R (1993). A social science appraoch to strenghening India's national tuberculsois programme. Indian J TB.

[65] World Health Organization. Addressing Poverty in TB Control - Options for National TB Control Programmes. World Health. 2005;4–80.

[66] Grede N, Claros JM, de Pee S, Bloem M (2014). Is there a need to mitigate the social and financial consequences of tuberculosis at the individual and household level?. AIDS Behav.

[67] Kamineni VV, Wilson N, Das A, Satyanarayana S, Chadha S, Singh Sachdeva K (2012). Addressing poverty through disease control programmes: examples from Tuberculosis control in India. Int J Equity Health.

