# Supplementary material for: Coping with tuberculosis and directly observed treatment: a qualitative study among patients from South India
Source: BMC Health Serv Res. 2016 Jul 19;16:283. doi: 10.1186/s12913-016-1545-9 (PMC4950693; doi:10.1186/s12913-016-1545-9)
Supplement: Additional file 1: — Interview guide 1. Patients under PPM-DOTS. Questionnaire for in-depth interviews of TB patients under PPM-DOTS (DOCX 29 kb) [file 12913_2016_1545_MOESM1_ESM.docx]

# Questionnaire for in-depth interviews of TB patients under PPM-DOTS

- The broad purpose of this questionnaire is to map pathways used by TB patients to navigate through health system to reach Public sector hospital- Designated Microscopic Centre (DMC) to get the sputum examination done for diagnosis of TB, followed by treatment
- Other objectives are to understand patient’s disease comprehension, experiences with health care providers (both public and private), agency in decision making process, their experiences of having DOTS from a PP, suggestions if any on improving access to TB diagnosis and treatment
- A brief outline of the objective and scope of the project and reasons for interviewing the respondent will be sent while seeking consent and fixing time for the interview. The schedule and place of the interview would be decided according to the convenience of the respondent to elicit good quality information

**Warming up**: (Introduction of researcher, explaining the objective of the project, obtain written consent and one or two general questions to make the respondent feel comfortable)

**General Information:**

TB number:

Date of interview: __/__/____

Place of interview:

Time interview started: ______________

Time interviewed completed:

1. **Health seeking behavior:**

- What were the initial complaints you had?
- How long it lasted?
- What did you do first?
- When did you seek treatment and why?
- Did you speak to somebody?
- Was it a problem for you?
- Please describe, since the beginning of developing symptoms to seeking care-which clinics/hospitals you visited over the time and why?

(Probe in detail to understand what factors affected the choice of particular healthcare providers over the time, look out for factors like fees, perceived popularity of providers, facility timings, providers’ attitudes, cultural/social linkages with providers, recommendations from family/friends etc.)

- What happened next? Did doctor advice any investigations? If so, where did you get the investigations done and what were those?
- Was there any delay or difficulty in getting your investigations done (probe for reasons for the delay and difficulties in terms of distance, transportation, etc)
- What was the experience in whole process of getting diagnosis done (probe for sputum examination experience)
- Cost incurred
- How do you feel about being diagnosed with TB?
- Did you accept the diagnosis or went for second opinion?
- **INITIATION OF TREATMENT:**
- Did you go back to the same doctor with lab results or went to different doctor, if so why?
- What did the doctor tell you looking at the lab results? Did he prescribe drugs immediately or after sometime? (What drugs, how many days) or referred you somewhere else? (If referred where and why?)
- How long you took the treatment of for TB? And where? (Any problems faced during this time like side effects, defaulted in between?)
- What was the cost incurred?
- When and why did you take a decision of going to Government hospital? (Recommendation by doctor or friends or relatives, influenced by media)
- What was your experience in DMC (who revealed results of the sputum test? Any IEC given during that time)
- What has been your experience with PP who is providing DOTS (convenient, perception of quality of care, attitude of PP, what are the advantages of taking DOTS from PP vis-à-vis from conventional DOT provider)

**B. Knowledge and beliefs about TB:**

- Were you aware of TB before you developed the disease?
- If yes, how? (TV/Print media/any family member suffered from TB)
- In your community, if someone has cough of long duration where do they access care (Public/private/quacks) and why?
- Do you think TB is a big problem in your community?
- If so, what is the usual response of the community when one gets diagnosed with TB? (Do friends and family members of TB patient worry about getting TB?)
- Do people with TB get concerned about being identified by others as someone who has TB?
- What do you think is the reason for contracting this disease in your case?
- Have you informed your family about your disease?
- If yes, what is their response to your disease? (Does your family accept your condition? does not accept and reasons for the same)

**(C) Past history of TB:**

- Were you treated for TB before?
- If yes, when, where and by whom?
- Daily or intermittent treatment? (Duration of treatment)
- Overall experience with diagnosis and treatment
- Did you experience any barriers to treatment (substance abuse, homelessness, lack of family support, and lack of finance?
- Do you have any associated medical conditions, including HIV/Diabetes/Hypertension/ Substance abuse? (Frequency, type, how long)

**(D) Treatment adherence:**

- What was your experience -right from diagnosis at DMC to DOTS provision?
- Are you satisfied with your DOTS treatment?
- Did you have to spend money to get DOTS TB treatment? If so how much and where you had to pay?
- How much money you paid for the PP who provided DOTS to you?
- Did you experience any side effects during DOTS?
- If so, whom did you consult? (It PP who was providing DOTS?)
- Were your concerns resolved?
- What do you think it would make it hard for a TB patient to get and adhere treatment? (Transportation, problem with time schedule, long waiting time, unfriendly hospital staff, substance abuse, homelessness, lack of finance, lack of family support, fear of drug side effects
- If the patient have had experience with both private and public sector, probe him to elaborate his experiences with treatment with DOTS vis a vis private treatment
- What was the total cost for diagnosis and treatment? (Did patient had to borrow money for this? if so how much and from whom?)
- Did you receive any financial support during the course of treatment (by Govt/NGO, etc.)?
- How can we ensure that TB sufferers do not stop taking TB treatment before completion of the course?

(E) **Personal information:**

Age: Caste/Tribe:

Place of residence:

Marital status:

Education: None Primary Secondary Tertiary

Number of children (Age and education):

Family type (Nuclear/Joint) and Family size (total family members sharing the same roof):

Type of house: Thatched house Mud house Brick house

Occupation:

Monthly income: None Rs.1000 –R2000 Rs.2000 – R3000 Rs.3000 - R4000 Rs.5000 and above

If unemployed, source of income:

Resident status (migrant, original) - How long you have been residing here?

- Do you have any question I need to answer?

If respondent says yes, answer respondents question, provide staff contact information for future questions, give out incentive and conclude interview.

If respondent says no, please provide staff contact information for future questions, give out incentive and conclude interview.

**Sources used to design questions:**

-R Shrestha-Kuwahara, M Wilce, HA Joseph, JW Carey, R Plank, and E Sumartojo. (2004):

Tuberculosis Research and Control; [www.findtbresources.org/material/Anthrop_Contrib.PDF](http://www.findtbresources.org/material/Anthrop_Contrib.PDF)

- Carolina Department of health services: <http://www.ctca.org/fileLibrary/file_69.pdf-Revised> edition of the expanded TB interview outline

-CDC TB information CD ROM-<http://www.tbchicago.org/tbguidecdc/ed_training/publications/ssmodules/ss6/ss6recordreview.htm>
